# Healthy Lifestyle Score and the Risk of Cardiovascular Disease, Cancer, and Mortality in an Asian Population: Prospective Cohort Study

**DOI:** 10.2196/95237

**Published:** 2026-11-11

**Authors:** Su Hyun Park, Peh Joo Ho, Xuan Liang, Rob van Dam, Mary F-F Chong, Charlie Guan Yi Lim, Xueling Sim, Airu Chia

**Affiliations:** 1 Saw Swee Hock School of Public Health, National University of Singapore, 12 Science Drive 2, #11-01, Singapore, Singapore, 117549, Singapore, 65 84361039; 2 George Washington University, Washington D.C., DC, United States

**Keywords:** healthy lifestyle score, mortality, acute myocardial infarction, AMI, stroke, cardiovascular disease, cancer

## Abstract

**Background:**

Healthy lifestyle behaviors are key modifiable factors for the prevention of chronic diseases and premature mortality. Composite lifestyle scores provide a practical framework for capturing the combined impact of multiple health behaviors, but prospective evidence from multiethnic Asian populations remains limited.

**Objective:**

This study examined the associations between a composite healthy lifestyle score and the risk of cardiovascular diseases, cancer, and all-cause mortality in a multiethnic Asian population.

**Methods:**

We analyzed prospective data from 9920 participants of the Singapore Multi-Ethnic Cohort who were free of major chronic diseases at baseline. A healthy lifestyle score was constructed from 5 components: BMI, smoking, physical activity, diet quality, and screen time. Scores ranged from 0 to 10, with higher scores reflecting healthier lifestyles. An expanded score, including mental well-being, was examined in a subset. Hazard ratios (HRs) and 95% CIs were estimated using the Cox proportional hazards model for acute myocardial infarction (AMI), stroke, cancer, and all-cause mortality, adjusting for sociodemographic factors, energy intake, family history of cancer or heart disease, and menopause status.

**Results:**

During a median follow-up of 14 years, 457 AMI cases, 322 stroke cases, 707 cancer incident cases, and 631 deaths were identified. More favorable lifestyle scores were associated with lower risk of cardiovascular outcomes and mortality, consistent with a dose-response relationship. Compared to participants scoring 8 to 10, those with the lowest score (0-3) had more than 2 times the risk of AMI (HR 2.13, 95% CI 1.47-3.07), stroke (HR 3.21, 95% CI 2.12-4.85), and all-cause mortality (HR 2.01, 95% CI 1.46-2.78). In contrast, no significant associations were observed for cancer incidence. Incorporating mental well-being into the lifestyle score yielded similar results.

**Conclusions:**

Adherence to a healthy lifestyle was associated with a lower risk of AMI, stroke, and mortality in a multiethnic Asian cohort, highlighting the importance of integrated lifestyle approaches for chronic disease prevention.

## Introduction

Noncommunicable diseases remain the leading cause of death worldwide, accounting for over 70% of mortality, with cardiovascular diseases (CVDs) causing most premature deaths, followed by cancers [1]. Key modifiable lifestyle factors such as smoking, physical inactivity, and unhealthy diets, along with metabolic risks such as overweight and obesity, which largely stem from these behaviors, are major contributors to the development of noncommunicable diseases [1,2].

Composite lifestyle scores that integrate multiple lifestyle factors into a single measure can provide a combined impact and holistic assessment of overall lifestyle [3]. Compared to individual risk factors, these composite scores show stronger associations with major chronic disease outcomes and all-cause mortality. Meta-analyses have reported that individuals with the healthiest composite lifestyle scores had a 55% lower risk of all-cause mortality than those with the worst lifestyle scores [4], while the risk reduction was 33% for physical activity alone [5] and 23% for diet alone [6]. This greater risk reduction likely reflects synergistic or multiplicative interactions among lifestyle behaviors, which are often not captured when individual factors are examined separately [7,8].

Despite robust evidence from Western populations [8,9], studies in Asian populations are comparatively fewer and largely based on East Asian cohorts in China, Japan, or South Korea [10-12]. This limits the generalizability of existing evidence to more diverse or multiethnic Asian contexts such as Singapore given differences in lifestyle behaviors across ethnic groups [13]. Prior cohort studies have rarely incorporated screen-based behavior or related health indicators such as mental well-being despite their relevance to broader lifestyle patterns. Excessive screen time has been associated with greater sedentary behavior, sleep disruption, and poorer mental well-being [14,15], which may collectively increase the risk of major cardiovascular outcomes and mortality [16-21]. A recent analysis of 13,282 adults aged 65 years and older in the Chinese Longitudinal Healthy Longevity Survey found that better subjective well-being (eg, less anxiety and greater happiness) was independently associated with a 14% lower risk of all-cause mortality and a 30% lower risk when combined with healthy lifestyle behaviors. However, the combined effects of lifestyle factors with mental well-being and screen time on major health outcomes have not been well examined in multiethnic Asian populations.

This study examined the association between a combined lifestyle score incorporating smoking, diet, physical activity, BMI, and screen time and major health outcomes, including all-cause mortality, stroke, acute myocardial infarction (AMI), and cancer, in a multiethnic population. Within individuals with available mental well-being data, the association between major health outcomes and the expanded lifestyle score including mental well-being was studied. In addition, we examined which individual lifestyle component contributed the most to the observed associations, thus providing insights into key targets for prevention and intervention.

## Methods

### Study Population

We used data from the Multi-Ethnic Cohort Phase 1 (MEC1), a population-based study of adult residents aged 21 years and older in Singapore that includes 3 major ethnic groups (Chinese, Indian, and Malay). MEC1 baseline data (T1) were collected between 2004 and 2010. Individuals with a history of cancer, CVD, stroke, renal failure, or serious mental illness at recruitment were excluded from cohort enrollment. The MEC1 baseline dataset was formed by combining 2 existing population-based studies conducted between 2004 and 2007: the Singapore Prospective Study Program and the Singapore Cardiovascular Cohort Study, with additional recruitment of participants from 2007 to 2010 (Singapore Consortium of Cohort Studies–Multi-Ethnic Cohort [SCCS-MEC]) through household visits and community outreach to achieve oversampling of Malay and Indian individuals in Singapore. All participants provided written informed consent, including consent for linkage with national disease registries. Detailed information on the cohort has been published previously [22].

Of the initial 12,988 participants, 12,384 (95.4%) remained after excluding those not from the 3 major ethnic groups (n=33), with missing interview data (n=42), or with preexisting CVD (n=328) or cancer (n=201). After further exclusions of those with missing lifestyle data (n=2376) or covariate data (n=19) and implausible energy intake (≤500 or ≥6000 kcal per day; n=69), 9920 participants were included in the final analysis (Multimedia Appendix 1). The median follow-up duration ranged from 14.1 years for cancer to 14.3 years for all-cause mortality, with 14.2 years for both AMI and stroke. Mental well-being data were available for 4791 participants from a subset of participants (Singapore Prospective Study Program and Singapore Cardiovascular Cohort Study) who completed the 36-item Short Form Health Survey version 2 (SF-36v2) emotional well-being items at baseline; therefore, analyses incorporating mental well-being were restricted to this subset. In the subset with mental well-being data, the median follow-up ranged from 16.1 years (AMI, stroke, and cancer) to 16.2 years (all-cause mortality).

### Construction of a Healthy Lifestyle Score

Six lifestyle factors were assessed at baseline: BMI, cigarette smoking, physical activity, diet quality, and screen time, with mental well-being available in a subset of the participants. BMI was calculated from measured height and weight, while the other factors were self-reported via an interviewer-administered questionnaire [22]. Each factor was categorized into 3 levels and assigned 0 to 2 points, with higher scores representing healthier behaviors. Three levels were chosen to balance the need to capture meaningful variation with the need to maintain sufficient sample size within each category.

BMI was classified using established Asian cutoffs, defined as less than 23.0 kg/m^2^ (2 points), 23.0 to 24.9 kg/m^2^ (1 point), and 25.0 kg/m^2^ or higher (0 points) [23,24]. Smoking status was categorized as “never” (2 points), “former” (1 point), or “current” (0 points). Leisure-time physical activity was assessed using a physical activity questionnaire [25] and expressed in metabolic equivalent of task (MET) hours per week. Participants were categorized into 7.5 or more MET hours per week (2 points), 0 to less than 7.5 MET hours per week (1 point), and none (0 points), consistent with the World Health Organization recommendation of 150 minutes or more of moderate-intensity physical activity per week [26]. Dietary intake was assessed using the validated semiquantitative food frequency questionnaire (FFQ) [27], and diet quality was evaluated using the Alternative Healthy Eating Index (AHEI) 2010 [19], with higher scores indicating better diet quality. Participants were categorized into tertiles: highest (2 points), intermediate (1 point), and lowest (0 points). Screen time was assessed through the amount of time spent watching television or playing computer or handheld video games daily. Participants were categorized into less than 2 hours per day (2 points), 2 to 4 hours per day (1 point), and more than 4 hours per day (0 points). In a subset of the population, mental well-being was assessed using the SF-36v2 emotional well-being subscale derived from 5 items assessing nervousness, low mood (being downhearted or depressed), calmness, and happiness over the previous 4 weeks, with higher scores indicating better mental well-being [28]. Scores were transformed into a norm-based T-score (mean 50, SD 10) following the SF-36v2 scoring manual [28]. Participants were categorized into tertiles: highest (2 points), intermediate (1 point), and lowest (0 points). As mental well-being data were available only for a subset of participants (n=4791), the main analyses were conducted using a 5-factor score in the entire cohort, while mental well-being was incorporated in additional analyses in the smaller subset.

The primary lifestyle score was calculated by summing all 5 factors, with possible scores ranging from 0 to 10. Participants were further grouped into 4 categories: score of 0 to 3 (least favorable lifestyle profile), score of 4 or 5, score of 6 or 7, and score of 8 to 10 (most favorable lifestyle profile; Table 1). In the subset with mental well-being data, an extended 6-factor lifestyle score was calculated by additionally including mental well-being, contributing scores from 0 to 2 to the previous 5-factor lifestyle score. This extended 6-factor score had a wider possible range from 0 to 12 and a different observed distribution. Participants were grouped separately into 4 ordered categories: scores of 1 to 4, 5 and 6, 7 and 8, and 9 to 12.

**Table 1.** Definition of the healthy lifestyle score.

Lifestyle factor and points	Description
BMI	
0	Obese (≥25.0 kg/m^2^)
1	Overweight (23.0‐24.9 kg/m^2^)
2	Normal (<23.0 kg/m^2^)
Cigarette smoking	
0	Current smoker
1	Former smoker
2	Never smoker
Physical activity	
0	None (0 MET^a^ h per wk)
1	Low (>0 to <7.5 MET h per wk)
2	Adequate (≥7.5 MET h per wk)
Diet quality	
0	Lowest tertile (poorest diet quality)
1	Middle tertile
2	Highest tertile
Screen time	
0	High (>4 h per d)
1	Moderate (2-4 h per d)
2	Low (<2 h per d)
Mental well-being	
0	Lowest tertile (poorest mental well-being)
1	Middle tertile
2	Highest tertile

a MET: metabolic equivalent of task.

### Outcome Ascertainment

Incident cases of AMI, stroke, all-site cancer, and deaths were ascertained through linkage with national disease registries maintained by the National Registry of Diseases Office, including the Singapore Myocardial Infarction Registry, Singapore Stroke Registry, Singapore Cancer Registry, and Singapore Death Registry.

Follow-up time was calculated from the date of baseline interview to the date of first occurrence of the outcome of interest, death, or end of follow-up (December 31, 2021), whichever came first.

### Covariates

Covariates included age, sex, ethnicity, educational level, income, total energy intake, family history of cancer or heart disease, and menopausal status in women. Age was categorized as 35 years or younger, 36 to 45 years, 46 to 55 years, and 56 years or older. Sex was defined as male or female. Ethnicity was classified as Chinese, Malay, or Indian. Educational attainment was grouped into 4 categories: primary or lower, secondary, postsecondary, and university or higher. Household income was categorized into predefined levels, with a separate category created for participants who refused to answer, did not know, or had missing information.

Total energy intake (kcal per day) was derived from the FFQ administered at baseline and modeled as a continuous variable. Family history of cancer or heart disease was coded as “yes” or “no” based on self-report. Menopausal status was classified as premenopausal or postmenopausal among women, and male participants were coded as a separate category to retain all participants in the model.

### Ethical Considerations

Ethics approval was obtained from the Institutional Review Board of National University of Singapore (NUS-IRB-2022-27). All participants provided written informed consent, including consent for linkage with national disease registries. Research data used for this secondary analysis were deidentified and key coded, with access restricted to authorized members of the research team.

### Statistical Analysis

Descriptive statistics were presented according to the categories of the lifestyle score. For continuous variables, *P* values were estimated by modeling the median value of each lifestyle score category as a continuous variable in linear regression. For categorical variables, *P* values were calculated using the Cochran-Armitage trend test. To assess potential selection bias related to missing lifestyle data, baseline characteristics were compared between participants included in the final analytic sample and those excluded because of missing lifestyle data.

Cox proportional hazards regression models were used to estimate hazard ratios (HRs) and 95% CIs for associations between the lifestyle score and each outcome, including incident AMI, incident stroke, incident cancer, and all-cause mortality. The proportionality assumption was assessed using Schoenfeld residuals; no violations were detected. Tests for linear trend were performed by modeling lifestyle score categories as a continuous variable in the regression model. All models were adjusted for age, sex, ethnicity, educational level, income, total energy intake, family history of cancer or heart disease, and menopausal status.

Additional analyses were conducted in the subset of participants with available mental well-being data by incorporating mental well-being as a sixth component of the lifestyle score. To facilitate comparison between the primary and extended lifestyle scores, both scores were also modeled as continuous variables, with HRs estimated per 1-point increase. Ethnicity-stratified analyses were performed, and multiplicative interaction terms between the lifestyle score and ethnicity were included in the Cox models to assess potential effect modification by ethnicity. To assess whether the associations were driven by any single component of the lifestyle score, leave-one-out analyses were performed by recalculating the lifestyle score after omitting one component at a time.

## Results

Differences between included participants and those excluded because of missing lifestyle data were observed for age, educational level, household income, total energy intake, and family history of cancer or heart disease, whereas sex, ethnicity, and menopausal status were similar between groups (Multimedia Appendix 2).

Table 2 shows the baseline characteristics of the participants according to the healthy lifestyle score. Of the 9920 participants, 4254 (42.9%) were male, and most (n=4480, 45.2%) were Chinese, followed by Indian (n=2790, 28.1%) and Malay (n=2650, 26.7%). Most participants (2923/9920, 29.5%) were aged 46 to 55 years. Participants with the least favorable lifestyle profile (score of 0‐3) were younger, more often male, and of non-Chinese ethnicity and had lower income and educational attainment (*P*<.01 in all cases). Compared with participants with the least favorable lifestyle profile, those in the highest category (score of 8‐10) had lower BMI (median 21.9, IQR 20.1-23.7 vs 27.4, IQR 25.3-30.3 kg/m^2^); comprised fewer smokers (18/2605, 0.7% vs 479/645, 74.3%); engaged in higher levels of leisure-time physical activity (median 13.6, IQR 8.0-24.5 vs 0.0, IQR 0.0-0.0 MET hours per week); and reported better diet quality (median AHEI score 55.8, IQR 50.5-60.2 vs 41.4, IQR 36.3-45.8), shorter screen time (median 0.0, IQR 0.5-1.5 vs 1.0, IQR 0.0-2.0 hours per day), and better mental well-being (mean T-score 50.9, SD 9.3 vs 48.9, SD 11.2; *P*<.001 in all cases). In contrast, total energy intake, menopausal status, and family history of cancer or heart disease did not differ significantly across categories.

**Table 2.** Baseline characteristics of study participants according to healthy lifestyle score (N=9920).^a^

	Total	Healthy lifestyle score				*P* value
		0-3 (n=645)	4-5 (n=2657)	6-7 (n=4013)	8-10 (n=2605)	
Age (y), n (%)						.01
≤35	2358 (23.8)	208 (32.2)	615 (23.1)	925 (23.1)	610 (23.4)	
36-45	2606 (26.3)	173 (26.8)	687 (25.9)	1043 (26)	703 (27)	
46-55	2923 (29.5)	163 (25.3)	848 (31.9)	1168 (29.1)	744 (28.6)	
≥56	2033 (20.5)	101 (15.7)	507 (19.1)	877 (21.9)	548 (21)	
Sex, n (%)						<.001
Male	4254 (42.9)	437 (67.8)	1227 (46.2)	1582 (39.4)	1008 (38.7)	
Female	5666 (57.1)	208 (32.2)	1430 (53.8)	2431 (60.6)	1597 (61.3)	
Ethnicity, n (%)						<.001
Chinese	4480 (45.2)	202 (31.3)	910 (34.2)	1833 (45.7)	1535 (58.9)	
Malay	2650 (26.7)	253 (39.2)	950 (35.8)	1027 (25.6)	420 (16.1)	
Indian	2790 (28.1)	190 (29.5)	797 (30)	1153 (28.7)	650 (25)	
Household monthly income (SGD; SGD 1=US $0.78 as of October 18, 2026), n (%)						<.001
<2000	2309 (23.3)	247 (38.3)	815 (30.7)	882 (22)	365 (14)	
2000-3999	2579 (26)	189 (29.3)	729 (27.4)	1073 (26.7)	588 (22.6)	
4000-5999	1624 (16.4)	68 (10.5)	385 (14.5)	654 (16.3)	517 (19.8)	
≥6000	1384 (14)	43 (6.7)	266 (10)	554 (13.8)	521 (20)	
Refused to answer or did not know	2024 (20.4)	98 (15.2)	462 (17.4)	850 (21.2)	614 (23.6)	
Educational level, n (%)						<.001
Primary or lower	2753 (27.8)	246 (38.1)	979 (36.8)	1086 (27.1)	442 (17)	
Secondary	3602 (36.3)	243 (37.7)	997 (37.5)	1484 (37)	878 (33.7)	
Postsecondary	2356 (23.8)	139 (21.6)	528 (19.9)	961 (23.9)	728 (27.9)	
University or higher	1209 (12.2)	17 (2.6)	153 (5.8)	482 (12)	557 (21.4)	
Total energy intake (kcal per d), median (IQR)	2111.8 (1651.7-2671.6)	2185.7 (1689.3-2829.0)	2094.2 (1618.1-2635.1)	5 (1625.7-2673.9)	2149.3 (1725.6-2670.5)	<.001
Family history of cancer or heart disease, n (%)						.16
No	7196 (72.5)	464 (71.9)	1903 (71.6)	2918 (72.7)	1911 (73.4)	
Yes	2724 (27.5)	181 (28.1)	754 (28.4)	1095 (27.3)	694 (26.6)	
Menopause status^b^, n/N (%)						.10
Premenopausal	3632/5666 (64.1)	143/208 (68.8)	895/1430 (62.6)	1533/2431 (63.1)	1061/1597 (66.4)	
Postmenopausal	2034/5666 (35.9)	65/208 (31.3)	535/1430 (37.4)	898/2431 (36.9)	536/1597 (33.6)	
BMI (kg/m^2^), median (IQR)	24.3 (21.5-27.7)	27.4 (25.3-30.3)	26.6 (24.1-29.6)	24.1 (21.4-27.2)	21.9 (20.1-23.7)	<.001
Smoking status, n (%)						<.001
Never	7657 (77.2)	108 (16.7)	1682 (63.3)	3369 (84)	2498 (95.9)	
Former	695 (7)	58 (9)	263 (9.9)	285 (7.1)	89 (3.4)	
Current	1568 (15.8)	479 (74.3)	712 (26.8)	359 (8.9)	18 (0.7)	
Screen time (h per d), median (IQR)	0.7 (0.0-2.0)	1.0 (0.0-3.0)	1.0 (0.0-2.0)	0.8 (0.0-2.0)	0.0 (0.5-1.5)	<.001
Physical activity (MET^c^ h per wk), median (IQR)	4.7 (0.0-15.0)	0.0 (0.0-0.0)	0.0 (0.0-5.3)	4.5 (0.0-14.2)	13.6 (8.0-24.5)	<.001
Diet quality (AHEI^d^ score), median (IQR)	50.1 (43.6-56.6)	41.4 (36.3-45.8)	45.4 (39.9-51.4)	50.6 (45.1-56.5)	55.8 (50.5-60.2)	<.001
Mental well-being (n=4791), mean (SD)	50.3 (10.0)	48.9 (11.2)	49.4 (10.8)	50.5 (9.8)	50.9 (9.3)	<.001

a *P* values were estimated by modeling the median value of each lifestyle score category as a continuous variable in linear regression for continuous outcomes and by using the Cochran-Armitage test for categorical outcomes.

b Only in women.

c MET: metabolic equivalent of task.

d AHEI: Alternative Healthy Eating Index.

The associations between the healthy lifestyle score and risk of health outcomes are shown in Table 3. Over a median follow-up of 14 years, 457 AMI cases, 322 stroke cases, 707 cancer cases, and 631 all-cause deaths were documented. In the adjusted model, participants with the least favorable lifestyle profile (score of 0‐3) had higher risks of AMI (HR 2.13, 95% CI 1.47‐3.07), stroke (HR 3.21, 95% CI 2.12‐4.85), and all-cause mortality (HR 2.01, 95% CI 1.46‐2.78) than those in the most favorable category (score of 8-10). Significant inverse dose-response trends were observed for these outcomes (*P*<.01 in all cases). No significant association was observed for cancer (HR 1.28, 95% CI 0.91‐1.79). Kaplan-Meier curves showed graded separation for mortality, AMI, and stroke, whereas cancer curves largely overlapped (Multimedia Appendix 3).

**Table 3.** Hazard ratios (HRs) for the associations between healthy lifestyle score and the risk of all-cause mortality, stroke, acute myocardial infarction (AMI), and cancer adjusting for age, sex, ethnicity, income, educational level, total energy intake, family history of cancer or heart disease, and menopause status.

Lifestyle scores	All-cause death (n=631; median follow-up 14.3 y)		AMI (n=457; median follow-up 14.2 y)		Stroke (n=322; median follow-up 14.2 y)		Cancer (n=707; median follow-up 14.1 y)	
	Cases, n	HR (95% CI)^a^	Cases, n	HR (95% CI)^a^	Cases, n	HR (95% CI)^a^	Cases, n	HR (95% CI)^b^
0-3	64	2.01 (1.46-2.78)	54	2.13 (1.47-3.07)	45	3.21 (2.12-4.85)	46	1.28 (0.91-1.79)
4-5	205	1.64 (1.28-2.09)	156	1.71 (1.28-2.28)	97	1.54 (1.09-2.18)	181	1.09 (0.87-1.35)
6-7	257	1.44 (1.14-1.81)	172	1.38 (1.05-1.81)	125	1.36 (0.99-1.88)	303	1.14 (0.95-1.38)
8-10	105	1.00	75	1.00	55	1.00	177	1.00

a *P*<.001.

b *P*=.24.

In analyses incorporating the mental health component into the lifestyle score, results were consistent. Participants with lower healthy lifestyle scores remained at higher risk of all-cause mortality and AMI, with similar estimates for these outcomes, while the association with stroke was slightly attenuated (HR 2.14, 95% CI 1.32‐3.46). No clear dose-response association was observed for overall cancer incidence (Multimedia Appendix 4).

When modeled as continuous variables, the primary lifestyle score and the score including mental well-being showed similar inverse associations with all-cause mortality, AMI, and stroke. The primary score was not associated with overall cancer incidence, whereas the score including mental well-being showed a modest inverse association with cancer (Multimedia Appendix 5).

Ethnicity-stratified analyses were conducted to assess potential effect modification by ethnicity. No significant interactions were observed for all-cause mortality, AMI, or stroke (*P*=.29, .41, and .43, respectively), whereas an interaction was observed for overall cancer incidence (*P*=.02). However, stratified analyses by ethnicity did not show significant dose-response trends within any ethnic group (Multimedia Appendix 6).

In leave-one-out analyses (Figure 1), exclusion of smoking attenuated the association with all-cause mortality (HR 1.46, 95% CI 0.85‐2.48), indicating that smoking was a major contributor to this association. For AMI, effect estimates remained largely significant across all exclusions, suggesting that the association was not driven by any single lifestyle component. For stroke, exclusion of smoking (HR 1.38, 95% CI 0.71‐2.67) or screen time (HR 1.57, 95% CI 0.89‐2.75) resulted in attenuation, indicating that these factors may play an important role in the observed associations. For cancer, the lifestyle score excluding physical activity was associated with risk (HR 1.56, 95% CI 1.02‐2.38), while exclusion of diet quality (AHEI score) showed a borderline significant association (HR 1.47, 95% CI 1.00‐2.15).

**Figure 1.**
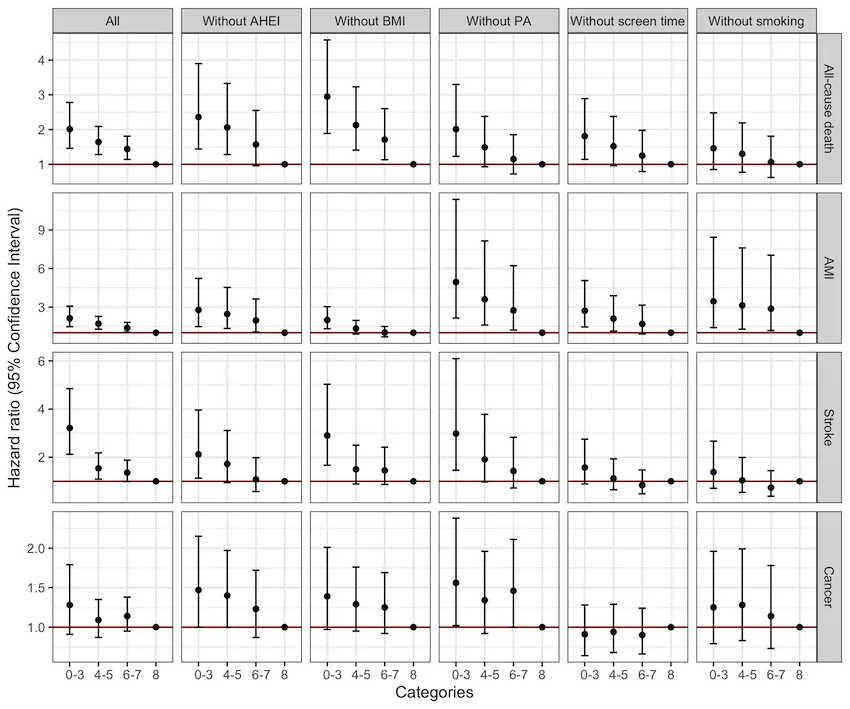
Leave-one-out analyses of the healthy lifestyle score in relation to major health outcomes. The healthy lifestyle score ranged from 0 to 10 in the full model and from 0 to 8 when an individual component was excluded. Higher scores indicate healthier lifestyle profiles. The points represent hazard ratios, and the error bars indicate 95% confidence intervals. AHEI: Alternative Healthy Eating Index; AMI: acute myocardial infarction; PA: physical activity.

## Discussion

### Principal Findings

In this multiethnic Asian cohort with over 14 years of follow-up, adherence to a healthy lifestyle as measured using a composite score was associated with substantially lower risks of all-cause mortality, AMI, and stroke. Participants with the least favorable lifestyle profiles had twice the risk of AMI and all-cause mortality and over 3 times the risk of stroke compared with those with the most favorable profiles. Results were consistent when mental well-being was incorporated in the lifestyle score. No significant associations were observed for overall cancer incidence. These findings highlight the importance of healthy lifestyle behaviors for cardiovascular health and longevity in Asian populations.

### Comparison With Previous Studies

Our findings are consistent with those of previous cohort studies reporting that composite healthy lifestyle scores are inversely associated with mortality and CVD outcomes in both Western and Asian populations [29]. Large cohorts from Europe and the United States have demonstrated that combinations of nonsmoking, healthy body weight, regular physical activity, light-to-moderate alcohol consumption, and high-quality diet are associated with reductions in all-cause and cardiovascular mortality [30,31]. Comparable associations have been documented in East Asian cohorts, including China, Taiwan, South Korea, and Japan, where higher lifestyle scores incorporating BMI, smoking, physical activity, diet, and alcohol consumption were observed [32-35]. In our study, participants with the least favorable lifestyle profiles had approximately 2.0 times the risk of all-cause mortality, 2.1 times the risk of AMI, and 3.2 times the risk of stroke, broadly consistent with meta-analyses of prospective cohort studies reporting 2.2 times higher all-cause mortality and 2.2 to 3.4 times higher risk of various cardiovascular outcomes [4]. Our study extends this literature to a multiethnic Southeast Asian population and incorporates screen-based sedentary behavior, assessed as television viewing and computer or handheld video game use, into the composite lifestyle score.

### Interpretation and Implications

A notable contribution of this study is the integration of screen time into the composite lifestyle score. Sedentary behavior, including prolonged screen time, has been increasingly recognized as an independent risk factor for cardiometabolic diseases. Epidemiological evidence across multiple cohorts has shown that increased leisure screen time is associated with greater cardiometabolic risk in both adolescents and adults, as well as with elevated risks of coronary artery disease and mortality [21,36,37]. Consistent with these findings, our leave-one-out analysis showed that removing screen time from the score substantially attenuated the association with stroke, suggesting that screen time may be particularly relevant for cerebrovascular risk. Potential mechanisms include impaired circulation, central adiposity, dysregulated glucose metabolism, elevated blood pressure, and inflammation, all of which are closely linked to ischemic stroke [38-41].

Although there is accumulating evidence suggesting that psychological distress and low mental well-being are associated with higher risks of mortality and CVDs [42-44], mental well-being has rarely been incorporated into lifestyle indexes. While mental well-being is not a lifestyle behavior per se, it is largely shaped by them, and including them in the combined lifestyle score allows for a broader assessment of health. In our study, mental well-being was assessed using the SF-36v2 mental health subscale, which has been used to predict mortality and CVD outcomes in prior prospective cohorts [45]. In the subgroup with available data, adding mental well-being to the score did not substantially change the overall patterns of association. The estimates remained similar for all-cause mortality and slightly attenuated for AMI and stroke but were statistically significant despite the smaller sample size. These findings suggest that mental well-being represents a complementary domain of lifestyle assessment, capturing psychosocial aspects of health that may support overall disease prevention and well-being.

The composite lifestyle score was designed to capture the cumulative influence of multiple lifestyle-related risk factors that commonly co-occur in real-world settings as compared to considering each lifestyle component independently. Smoking, poor diet quality, lower physical activity, and sedentary behaviors may cluster within broader unhealthy lifestyle patterns, and the score provides a practical summary measure of this overall lifestyle risk. In sensitivity analyses excluding smoking, the associations with all-cause mortality and stroke were attenuated and no longer statistically significant, suggesting that smoking was a particularly influential component for these outcomes. This pattern aligns with extensive evidence showing that smoking promotes endothelial dysfunction, systemic inflammation, oxidative stress, and thrombotic pathways that accelerate atherosclerosis and increase risks of ischemic stroke and premature death [46-49]. These findings highlight the importance of smoking prevention and cessation for reducing the risks of premature mortality and stroke.

There was no significant evidence of interaction by ethnicity for the associations with all-cause mortality, AMI, and stroke. Although an interaction was observed for overall cancer incidence, the ethnicity-stratified analyses did not show clear dose-response trends. This finding should be interpreted cautiously. The null association between the lifestyle score and overall cancer incidence should also be interpreted with caution. Cancer encompasses multiple diseases with distinct etiologies and typically long latency periods, and aggregating all cancer types into a single outcome may obscure lifestyle-related associations that differ by cancer site. Furthermore, the relatively young baseline age of our cohort and comparatively short follow-up for cancer outcomes resulted in a limited number of cancer events during follow-up, reducing the power to detect associations.

### Limitations

This study has several limitations. First, the lifestyle score was constructed as an unweighted index summing all lifestyle components equally and assuming each factor had equal importance. Previous studies have examined both weighted and unweighted indexes, showing that both effectively predict CVD risk [50]. The unweighted index is outcome agnostic, which is more practical and can be applied to a broader range of health outcomes. Second, lifestyle behaviors were primarily self-reported, which may introduce recall error or reporting bias. However, such errors are likely nondifferential as outcomes were assessed prospectively. The physical activity questionnaire and FFQ have been validated in independent samples of the local population with similar characteristics [22,27,51]. In addition, differences in several baseline characteristics between included participants and those excluded because of missing lifestyle data may have introduced selection bias. Third, lifestyle behaviors and covariates were measured at baseline only, and changes in these behaviors during follow-up may have introduced nondifferential misclassification that biased the association toward the null. Fourth, alcohol consumption was not included as a separate lifestyle component as it was already incorporated into the AHEI 2010 score to avoid double counting. Fifth, although we adjusted for multiple confounders, our findings could still be influenced by residual confounding and unmeasured factors. While the median follow-up of approximately 14 years allowed for assessment of long-term outcomes, longer follow-up may be needed to further clarify associations, particularly for cancer outcomes with longer latency. Sixth, cancer was analyzed as a single combined end point. Although overall cancer incidence provides a broad assessment of cancer burden alongside other major chronic disease outcomes, cancer comprises a heterogeneous group of diseases with distinct etiologies, and combining all incident cancers may obscure site-specific associations with lifestyle factors. Therefore, findings for overall cancer incidence should be interpreted cautiously. Finally, as this study was conducted in a multiethnic Asian cohort, the findings may not be generalizable to other populations with different sociodemographic or cultural contexts.

### Conclusions

Our findings highlight the importance of combined lifestyle behaviors, including screen-based sedentary behavior, for cardiovascular and overall health. Incorporating mental well-being yielded similar results, suggesting that a broader conceptualization of lifestyle may be relevant for chronic disease prevention. These results highlight opportunities for comprehensive lifestyle interventions and public health strategies to reduce CVD and premature mortality in Asian populations.

## Supplementary material

10.2196/95237 Multimedia Appendix 1 Study flowchart.

10.2196/95237 Multimedia Appendix 2 Baseline characteristics of participants included in the final analytic sample and those excluded because of missing lifestyle data.

10.2196/95237 Multimedia Appendix 3 Kaplan-Meier curves for all-cause death, acute myocardial infarction, stroke, and cancer incidence.

10.2196/95237 Multimedia Appendix 4 Associations between healthy lifestyle score (including mental well-being) and all-cause mortality, stroke, acute myocardial infarction, and cancer (n=4791).

10.2196/95237 Multimedia Appendix 5 Associations between both the primary lifestyle score and the lifestyle score including mental well-being and all-cause mortality, acute myocardial infarction, stroke, and overall cancer incidence.

10.2196/95237 Multimedia Appendix 6 Associations between the healthy lifestyle score and all-cause mortality, acute myocardial infarction, stroke, and cancer stratified by ethnicity.

10.2196/95237 Checklist 1 STROBE checklist.

## Abbreviations

AHEI: Alternative Healthy Eating Index
AMI: acute myocardial infarction
CVD: cardiovascular disease
FFQ: food frequency questionnaire
HR: hazard ratio
MEC1: Multi-Ethnic Cohort Phase 1
MET: metabolic equivalent of task
SCCS–MEC: Singapore Consortium of Cohort Studies–Multi-Ethnic Cohort
SF-36v2: 36-item Short Form Health Survey version 2
STROBE: Strengthening the Reporting of Observational Studies in Epidemiology
