# Supplementary material for: Healthy Lifestyle Score and the Risk of Cardiovascular Disease, Cancer, and Mortality in an Asian Population: Prospective Cohort Study
Source: JMIR Public Health Surveill. 2026 Nov 11;12:e95237. doi: 10.2196/95237 (PMC13754259; doi:10.2196/95237)
Supplement: Supplementary file 1 [file publichealth-v12-e95237-s001.docx]

Multimedia Appendix 1. Study flowchart

| MEC1 Phase 1 linked with the National Registry of Diseases Office (NRDO) databases, including the Singapore Cancer Registry (SCR), Stroke Registry, Myocardial Infarction Registry, and Registry of Births and Deaths  (N=12,988) | |  |  |
| --- | --- | --- | --- |
|  |  |  | - Records with ethnicity “other” removed (n=33)  - Missing interview date excluded (n=42) |
|  |  |  |  |
| N=12,913 | |  |  |
|  |  |  |  |
|  |  |  | - Heart disease, heart attack, angioplasty/ballooning or heart bypass surgery removed (n=227) |
|  |  |  | - Stroke or transient ischemic attack (TIA/mini-stroke) removed (n=101) |
|  |  |  | - Cancer prevalent cases removed (n=201) |
| Eligible participants  (N=12,384) | |  |  |
|  |  |  | - Missing data on lifestyle variables excluded (n=2,376) |
|  |  |  | - Implausible energy intake (≤500kcal/day or ≥6,000kcal/day) excluded (n=69) |
|  |  |  |  |
|  |  |  | - Missing education (n=5) |
|  |  |  | - Missing menopausal status (n=14) |
| Eligible participants with the complete set of variables included in the analyses (N=9,920) | |  |  |
