# Supplementary material for: Healthy Lifestyle Score and the Risk of Cardiovascular Disease, Cancer, and Mortality in an Asian Population: Prospective Cohort Study
Source: JMIR Public Health Surveill. 2026 Nov 11;12:e95237. doi: 10.2196/95237 (PMC13754259; doi:10.2196/95237)
Supplement: Supplementary file 2 [file publichealth-v12-e95237-s002.docx]

Multimedia Appendix 2. Baseline characteristics of participants included in the final analytic sample and those excluded because of missing lifestyle data.

|  | Included (n=9,920) | Excluded-missing lifestyle variables  (n=2,340) | P-value |
| --- | --- | --- | --- |
| Age, cont | 45.23 (0.13) | 45.88 (0.29) | 0.03 |
| Age |  |  |  |
| <=35 | 2,358 (23.8) | 620 (26.5) | <0.0001 |
| 36-45 | 2,606 (26.3) | 604 (25.8) |  |
| 46-55 | 2,923 (29.5) | 529 (22.6) |  |
| 56 or older | 2,033 (20.5) | 587 (25.1) |  |
| Sex |  |  |  |
| Male | 4,254 (42.9) | 1,038 (44.4) | 0.195 |
| Female | 5,666 (57.1) | 1,302 (55.6) |  |
| Ethnicity |  |  |  |
| Chinese | 4,480 (45.2) | 1,035 (44.2) | 0.365 |
| Malay | 2,650 (26.7) | 659 (28.2) |  |
| Indian | 2,790 (28.1) | 646 (27.6) |  |
| Income |  |  |  |
| <2,000 | 2,309 (23.3) | 525 (22.4) | <0.0001 |
| 2000-3999 | 2,579 (26.0) | 513 (21.9) |  |
| 4000-5999 | 1,624 (16.4) | 249 (10.6) |  |
| 6000 or more | 1,384 (14.0) | 227 (9.7) |  |
| Unknown | 2,024 (20.4) | 826 (35.3) |  |
| Education |  |  |  |
| No formal | 730 (7.4) | 314 (13.4) | <0.0001 |
| Primary | 2,023 (20.4) | 549 (23.5) |  |
| Secondary | 3,602 (36.3) | 721 (30.8) |  |
| ITE/NTC | 621 (6.3) | 146 (6.2) |  |
| A level/polytec | 1,735 (17.5) | 344 (14.7) |  |
| University | 1,209 (12.2) | 266 (11.4) |  |
| Total energy intake | 2,246.11 (8.41) | 2,090.70 (17.58) | <0.0001 |
| Family history of cancer or heart diseases |  |  |  |
| No | 7,196 (72.5) | 1,893 (80.9) | <0.0001 |
| Yes | 2,724 (27.5) | 447 (19.1) |  |
| Menopause status  (only in women N=7034) |  |  |  |
| Premenopausal | 3,632 (64.1) | 804 (61.8) | 0.112 |
| Postmenopausal | 2,034 (35.9) | 498 (38.3) |  |
