# Supplementary material for: Healthy Lifestyle Score and the Risk of Cardiovascular Disease, Cancer, and Mortality in an Asian Population: Prospective Cohort Study
Source: JMIR Public Health Surveill. 2026 Nov 11;12:e95237. doi: 10.2196/95237 (PMC13754259; doi:10.2196/95237)
Supplement: Supplementary file 3 [file publichealth-v12-e95237-s003.docx]

|  |  |
| --- | --- |
|  |  |

Multimedia Appendix 3. Kaplan-Meier curves for all-cause death, acute myocardial infarction (AMI), stroke and cancer incidence
