# Supplementary material for: Healthy Lifestyle Score and the Risk of Cardiovascular Disease, Cancer, and Mortality in an Asian Population: Prospective Cohort Study
Source: JMIR Public Health Surveill. 2026 Nov 11;12:e95237. doi: 10.2196/95237 (PMC13754259; doi:10.2196/95237)
Supplement: Supplementary file 4 [file publichealth-v12-e95237-s004.docx]

Multimedia Appendix 4. Associations of healthy lifestyle score (with mental wellbeing) with all-cause mortality, stroke, AMI and cancer (N=4,791)

|  | All-cause death (N=388)  Median fu years=16.2years | | AMI (N=239)  Median fu years=16.1years | | Stroke (N=189)  Median fu years=16.1 years | | Cancer (N=451)  Median fu years=16.1years | |
| --- | --- | --- | --- | --- | --- | --- | --- | --- |
|  | Cases | HR (95% CI) | Cases | HR (95% CI) | Cases | HR (95% CI) | Cases | HR (95% CI) |
| **Lifestyle score** |  |  |  |  |  |  |  |  |
| 1-4 (n=411) | 49 | 2.01 (1.38, 2.94)** | 34 | 2.09 (1.32, 3.33)** | 31 | 2.14 (1.32, 3.46)** | 36 | 1.20 (0.82, 1.78) |
| 5-6 (n=1,197) | 111 | 1.59 (1.16. 2.17)** | 70 | 1.59 (1.08, 2.35)* | 50 | 1.16 (0.76, 1.76) | 116 | 1.26 (0.96, 1.66) |
| 7-8 (n=1,862) | 161 | 1.58 (1.18, 2.11)** | 92 | 1.53 (1.06, 2.20)* | 65 | 1.03 (0.70, 1.52) | 192 | 1.33 (1.05, 1.69)* |
| 9-12 (n=1,321) | 67 | Ref | 43 | Ref | 43 | Ref | 107 | Ref |
| p-trend |  | 0.001 |  | 0.002 |  | 0.007 |  | 0.93 |

Adjusting for age, gender, ethnicity, income, education, total energy intake, family history of cancer or heart diseases, and menopausal status

*p<0.05; **p<0.01
