# Supplementary material for: Healthy Lifestyle Score and the Risk of Cardiovascular Disease, Cancer, and Mortality in an Asian Population: Prospective Cohort Study
Source: JMIR Public Health Surveill. 2026 Nov 11;12:e95237. doi: 10.2196/95237 (PMC13754259; doi:10.2196/95237)
Supplement: Supplementary file 5 [file publichealth-v12-e95237-s005.docx]

Multimedia Appendix 5. Associations of the primary lifestyle score and the lifestyle score including mental wellbeing with all-cause mortality, AMI, stroke, and overall cancer incidence.

|  | Death | AMI | Stroke | Cancer |
| --- | --- | --- | --- | --- |
|  | HR (95% CI) | HR (95% CI) | HR (95% CI) | HR (95% CI) |
| Primary 5-factor score, full cohort  N=9,920 | 0.89 (0.85, 0.93)**  N=631 | 0.88 (0.83, 0.93)**  N=457 | 0.86 (0.80, 0.91)**  N=322 | 0.98 (0.94, 1.03)  N=707 |
| 6-factor score including mental wellbeing, subset  N=4,791 | 0.91 (0.86, 0.95)**  N=388 | 0.89 (0.83, 0.95)**  N=239 | 0.91 (0.84, 0.98)*  N=189 | 0.95 (0.90, 1.00)*  N=451 |

Adjusting for age, gender, ethnicity, income, education, total energy intake, family history of cancer or heart diseases, and menopausal status.

*p<0.05; **p<0.01
