# Supplementary material for: Healthy Lifestyle Score and the Risk of Cardiovascular Disease, Cancer, and Mortality in an Asian Population: Prospective Cohort Study
Source: JMIR Public Health Surveill. 2026 Nov 11;12:e95237. doi: 10.2196/95237 (PMC13754259; doi:10.2196/95237)
Supplement: Supplementary file 6 [file publichealth-v12-e95237-s006.docx]

Multimedia Appendix 6. Associations of the healthy lifestyle score with all-cause mortality, AMI, stroke, and cancer, stratified by ethnicity

|  |  | Death | | AMI | | Stroke | | Cancer | |
| --- | --- | --- | --- | --- | --- | --- | --- | --- | --- |
| Chinese | Lifestyle  scores | Cases | HR (95% CI) | Cases | HR (95% CI) | Cases | HR (95% CI) | Cases | HR (95% CI) |
|  | 0-3 | 17 | 1.60 (0.92, 2.79) | 19 | 3.91 (2.16, 7.07)** | 13 | 2.62 (1.34, 5.15)** | 15 | 1.13 (0.65, 1.97) |
|  | 4-5 | 65 | 1.52 (1.05, 2.19)* | 37 | 1.73 (1.06, 2.82)* | 28 | 1.36 (0.80, 2.31) | 82 | 1.34 (0.99, 1.81) |
|  | 6-7 | 114 | 1.47 (1.06, 2.02)* | 54 | 1.38 (0.89, 2.14) | 60 | 1.54 (0.99, 2.40) | 175 | 1.40 (1.09, 1.79)** |
|  | 8-10 | 57 | Ref | 32 | Ref | 30 | Ref | 104 | Ref |
|  | p-trend |  | 0.02 |  | <0.0001 |  | 0.024 |  | 0.105 |
| Malay | Lifestyle  scores | Cases | HR (95% CI) | Cases | HR (95% CI) | Cases | HR (95% CI) | Cases | HR (95% CI) |
|  | 0-3 | 24 | 1.85 (1.01, 3.38)* | 22 | 2.01 (1.01, 4.02)* | 14 | 2.46 (1.08, 5.57)* | 18 | 1.01 (0.56, 1.83) |
|  | 4-5 | 85 | 1.67 (1.03, 2.72)* | 59 | 1.58 (0.87, 2.87) | 39 | 1.51 (0.76, 2.99) | 49 | 0.61 (0.39, 0.96)* |
|  | 6-7 | 66 | 1.08 (0.66, 1.77) | 43 | 1.08 (0.59, 1.98) | 35 | 1.12 (0.57, 2.23) | 66 | 0.69 (0.46, 1.05) |
|  | 8-10 | 21 | Ref | 14 | Ref | 11 | Ref | 36 | Ref |
|  | p-trend |  | 0.002 |  | 0.008 |  | 0.02 |  | 0.39 |
| Indian | Lifestyle  scores | Cases | HR (95% CI) | Cases | HR (95% CI) | Cases | HR (95% CI) | Cases | HR (95% CI) |
|  | 0-3 | 23 | 2.47 (1.36, 4.46)** | 13 | 1.23 (0.62, 2.44) | 18 | 4.77 (2.25, 10.12)** | 13 | 1.29 (0.66, 2.52) |
|  | 4-5 | 55 | 1.59 (0.98, 2.56) | 60 | 1.62 (1.02, 2.57)* | 30 | 1.65 (0.86, 3.20) | 50 | 0.99 (0.63, 1.54) |
|  | 6-7 | 77 | 1.75 (1.12, 2.74)* | 75 | 1.64 (1.06, 2.54)* | 30 | 1.24 (0.65, 2.36) | 62 | 0.92 (0.61, 1.38) |
|  | 8-10 | 27 | Ref | 29 | Ref | 14 | Ref | 37 | Ref |
|  | p-trend |  | 0.01 |  | 0.25 |  | <0.0001 |  | 0.62 |
|  | p-interaction | 0.29 | | 0.41 | | 0.43 | | 0.02 | |

Adjusting for age, gender, income, education, total energy intake, family history of cancer or heart diseases, and menopausal status.
